# Use of miglustat in a child with late-infantile-onset Niemann-Pick disease type C and frequent seizures: a case report

**DOI:** 10.1186/1752-1947-6-383

**Published:** 2012-11-12

**Authors:** Johannes Skorpen, Ingrid B Helland, Bjørn Tennøe

**Affiliations:** 1 Department of Paediatric Medicine, Ålesund Hospital, Child Habilitation Unit, N-6026, Ålesund, Norway; 2 Children’s Department, Oslo university Hospital, Rikshospitalet, P O Box 4950, Nydalen, NO-0424, Oslo, Norway; 3 Department of Radiology, Oslo university Hospital, Rikshospitalet, P O Box 4950, Nydalen, NO-0424, Oslo, Norway

**Keywords:** Niemann-Pick disease type C, Infant, Miglustat, Treatment, Seizures, Anti-epileptic

## Abstract

**Introduction:**

Niemann-Pick disease type C is a rare genetic lysosomal storage disease associated with impaired intracellular lipid trafficking and a range of progressive neurological manifestations. The influence of seizure activity on disease course and response to miglustat therapy is not currently clear.

**Case presentation:**

Niemann-Pick disease type C homozygous for *NPC1* mutation p.S940L [c. 2819 C>T] was diagnosed in a four-and-a-half-year-old Norwegian Caucasian girl. The patient, who died at eight years and seven months of age, had a history of prolonged neonatal jaundice and subsequently displayed progressive neurological manifestations that started with delayed speech, ataxia, and gelastic cataplexy. A regimen of 100mg of miglustat three times a day was initiated when she was four years and 11 months old. She showed decreased neurological deterioration during about three and a half years of treatment. However, she displayed periods of distinct worsening that coincided with frequent epileptic seizures. Anti-epileptic therapy reduced seizure frequency and severity and allowed re-stabilization of her neurological function. Prior to her death, which was possibly due to acute cardiac arrest, seizure activity was well controlled.

**Conclusions:**

Miglustat delayed the expected deterioration of neurological function in this patient with p.S940L-homozygous late-infantile-onset Niemann-Pick disease type C and provided important quality-of-life benefits. This case demonstrates the importance of effective seizure control therapy in achieving and maintaining neurological stabilization in Niemann-Pick disease type C.

## Introduction

Niemann-Pick disease type C (NP-C) is a potentially devastating progressive neurodegenerative disease currently estimated to occur in 1:100,000 to 1:120,000 live births [1]. NP-C is caused by autosomal recessive mutations in both alleles of either the *NPC1* gene, which is detected in 95% of cases, or the *NPC2* gene [2]. These mutations give rise to impaired intracellular lipid trafficking and subsequent accumulation of unesterified cholesterol, sphingosine, and a range of glycosphingolipids in various tissues, including the brain [1].

NP-C has an extremely heterogeneous clinical presentation characterized by a wide range of systemic, neurological, and psychiatric symptoms, many of which are not specific to the disease [3]. This makes it difficult to establish an early diagnosis. Patients may present during infancy, but many cases present during adolescence or adulthood [1,3]. Clinical NP-C phenotypes can be broadly defined on the basis of age at disease onset [3].

Until recently, no disease-modifying therapy was available for NP-C. In 2009, miglustat (Zavesca, Actelion Pharmaceuticals Ltd., Allschwil, Switzerland) was approved in Europe for the treatment of adults and children with NP-C on the basis of clinical trial data and a retrospective observational cohort study showing improvements or stabilization of neurological disease manifestations [4-7]. To date, no published reports have assessed the influence of seizure activity on disease course in NP-C or the possible impact of seizures on patient responses to miglustat therapy. We report the case of a young patient who had late-infantile NP-C and significant seizure activity and was treated with miglustat and anti-epileptic therapy.

## Case presentation

The patient was a girl born to Norwegian Caucasian parents following a normal pregnancy and birth; her birth weight was 3.44kg. As a neonate, she had jaundice that persisted for three months and that was considered to be due to breast feeding/breast milk icterus. No clinical signs of hepatomegaly or splenomegaly were noted, and further investigations, including abdominal ultrasound, were not performed. After an initial assessment, she showed normal healthy psychomotor development and was walking independently at 12 months of age.

Follow-up was initiated locally when she was about two and a half years old because her speech development was delayed. When referred to the pediatric department at the age of two years and 11 months, she showed slowing of motor development, impaired balance and coordination, episodes of gelastic cataplexy, and arrested language and psychomotor development. Findings from brain magnetic resonance imaging (MRI), electroencephalogram, urine/plasma metabolic screens, and cerebrospinal fluid analyses were all normal. Clinical examination revealed no signs of hepatosplenomegaly.

She developed swallowing difficulties at three years and four months, at which time her fine motor skills had deteriorated further and included visible tremor. Vertical gaze palsy and ataxia were detected two months later, and her cataplectic episodes continued. Language testing showed pronounced deficits in speech. By the age of four years, her swallowing difficulties had noticeably worsened and she was losing further fine and gross motor skills, commencing the use of a walker to ambulate indoors and a wheelchair for outdoor ambulation. Her cognitive function was also significantly impaired, and repeat language testing indicated ongoing deterioration in speech. MRI analysis at the age of four years and seven months showed deep bilateral cerebral white-matter signal hyperintensities – a leukodystrophy-like pathology.

The rapidly deteriorating disease prompted a search for a specific diagnosis. The combination of cataplectic episodes, progressive ataxia, and a notable vertical gaze palsy ultimately provoked suspicion of possible late-infantile NP-C. When she was four years and eight months old, a diagnosis of NP-C was confirmed on the basis of filipin staining and cholesterol esterification assay findings. Genetic analysis later that year showed homozygous p.S940L (c. 2819 C>T) mutations in the *NPC1* gene. Both parents were heterozygous for this mutant allele.

A regimen of 100mg of miglustat three times a day was commenced when our patient was four years and 11 months old, and regular follow-up was conducted until her death at the age of eight years and seven months; the total treatment period was three years and eight months. Before initiation of miglustat, a low-carbohydrate diet was implemented to reduce potential gastrointestinal disturbances. This diet was gradually phased out after four months of therapy. No gastrointestinal disturbances have been reported during miglustat therapy, possibly because of the early implementation of this low-carbohydrate diet.

At follow-up after one month on miglustat, our patient, who was five years old, showed continued deterioration in motor function in comparison with findings six months earlier. She was increasingly tired, and signs of spasticity were developing in her lower extremities. However, she displayed a noticeable improvement in chewing and swallowing function, and her overall awareness and concentration were improved.

Follow-up after six months on miglustat detected a general improvement in motor function. At 13-month follow-up, when she was six years old, her sitting balance and posture and both indoor and outdoor assisted ambulation were improved. Her gaze was also more stable, and she was continent for both urine and feces – a new skill. She no longer coughed or choked while drinking water. However, she showed gradually less interest in food, and it was decided that a gastrostomy tube should be established.

A general loss of energy and overall function that coincided with an increased frequency of epileptic attacks was observed about two and a half years ago. Concerted anti-epileptic treatment, which is described in detail below, significantly reduced the number of epileptic episodes, and she gradually improved to become medically stable. Although her functional abilities were variable, she was generally continent and was eating, drinking, sitting, and ambulating well. Two years ago, when she was seven years old, she experienced another period of frequent epileptic attacks and concurrent breathing difficulties with massive overproduction of mucus. As a result, she was temporarily hospitalized and during that time she again displayed a generalized deterioration in function. Control of her seizures was re-established about two months later, and anti-asthmatic therapy was reinforced. Within a short time, she showed signs of recovery and had retained her cognitive function.

**Figure 1:**
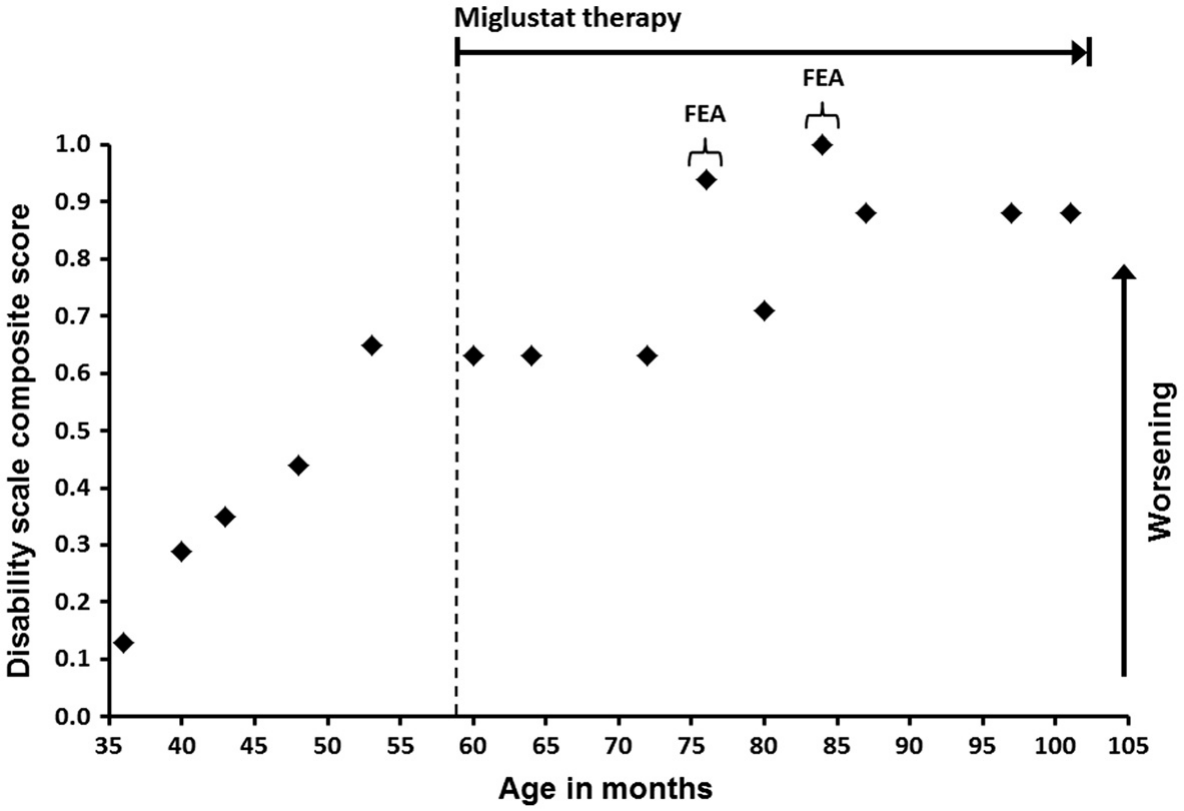
**Composite Niemann-Pick disease type C disability scale score during about five and a half years of follow-up.** Composite scores on the modified Niemann-Pick disease type C disability scale [7] are rated from zero, indicating the best, to one, indicating the worst. Disability scale assessments were performed by the author together with a child physiotherapist during planned follow-up visits at hospital from when the patient was six years and nine months old, to when she was eight years and five months old. Disability scores before age six years and nine months were evaluated retrospectively on the basis of patient records. FEA: frequent epileptic attacks.

Her function across four key parameters of neurological disease progression in NP-C – ambulation, manipulation, language, and swallowing – was assessed by using a published disease-specific disability scale modified to rate function across each domain from zero, indicating the best, to one, indicating the worst [7]. Disability assessments were undertaken at planned visits approximately every six months from the time of our first data collection two years ago and retrospectively at time points before that. Overall, data from disability scale assessments were available between the ages of three years and eight and a half years for a total follow-up period of five and a half years and reflected the changes recorded on the basis of empirical clinical observations (Figure 1).

Composite disability scores indicated sequential worsening during the 17 months before initiation of miglustat therapy, increasing from 0.13 when our patient was 36 months old to 0.65 at the last pretreatment assessment, when she was about four and a half years old. After miglustat was started, scores leveled off for about 17 months, remaining at 0.63 when she was five years old up to the first period of frequent epileptic attacks, when she was about six and a half years old, after which there was some worsening (Figure 1). Assessment four months later indicated a return toward stable disease, with a score of 0.71. Overall, these changes were dictated mainly by ambulation, manipulation, and swallowing function; speech had reached the near-maximal degree of impairment measurable before miglustat was started. During the second period of increased epileptic activity, when she was seven years old (Figure 1), her composite disability score increased to the maximum level of 1, indicating severe disability across all subscores. Her ambulation and language scores fell back to 0.88 when her epileptic episodes were once again under control three months later, but her manipulation and swallowing disabilities remained at 1, indicating permanent losses of function. Her remaining swallowing function was used only for tasting of food, not for feeding. There was no change in disability scale scores during follow-up clinical assessments afterward.

During the last clinical assessment before her death, she was awake and alert and interested in her surroundings, family members, teachers, and friends. Her disability scale score remained stable. While her ambulation and non-verbal communication were less frequent, she still conducted these activities in a purposeful manner. She also retained some function in terms of simple manipulation movements such as touching and reaching, raising arms, and assisting in feeding. On two separate occasions, seven weeks and three weeks prior to her death, she was hospitalized because of acute lung infections, which were resolved with treatment. Her precise cause of death is not known. She had no prevailing breathing difficulties or mucus overproduction and no signs of ongoing infection. An acute cardiac arrest cannot be ruled out.

Throughout her care, a rigorous approach was required to control her cataplexy and seizure activity. Initial treatment of her cataplexy with a daily dose of 5mg of fluoxetine when she was four years and nine months old reduced the frequency and severity of episodes by about 70%. However, fluoxetine was stopped when anti-epileptic treatment with 0.125mg of clonazepam three times a day was commenced following the onset of epilepsy with a long-lasting generalized tonic-clonic seizure. After two months, clonazepam was replaced by levetiracetam at doses gradually increased from 200mg twice a day to 450mg twice a day, which provided adequate seizure control for about six months until the first period of frequent epileptic attacks about two and a half years ago. Control was re-established initially by using 3.75mg of oral nitrazepam per day followed by maintenance therapy with a combination of levetiracetam and 7.5 to 10mg of clobazam per day. After the second period of frequent epileptic attacks two years ago, control was re-established by adding 250mg of valproic acid twice a day to her existing combination therapy. After this, she was clinically seizure-free for more than a year. During the months prior to her death she had four to six seizures each week, lasting 10 to 30 seconds. But it was not considered that this activity required any alteration to her anti-epileptic therapy.

## Discussion

This case report concerns the first known patient diagnosed as being homozygous for the *NPC1* gene mutation, p.S940L. To the best of our knowledge, the clinical phenotype for homozygous p.S940L NP-C has not been described before. The course of progressive neurological symptoms in our patient, preceded by initial isolated neonatal jaundice, is in agreement with previous data on the natural history of late-infantile-onset NP-C [1,8,9]. However, the absence of hepatomegaly or splenomegaly in a patient with late-infantile onset is unusual. Although no specific ultrasound examinations were performed to assess the liver or spleen before diagnosis, repeated abdominal assessments since diagnosis showed only mildly increased spleen size. It cannot be stated for sure whether the absence of organomegaly represents a feature specific to homozygous p.S940L NP-C or whether the time window during which it was clinically measurable was missed.

In line with published data in childhood-onset NP-C, miglustat appeared effective in stabilizing neurological disease in our patient [6,8]. During about three and a half years on miglustat, her rate of neurological deterioration was notably slower compared with the steady progression observed before therapy. While her epilepsy and occasional serious respiratory infections led to periods of deterioration, she regained a number of her previous levels of function a number of times.

Disease stabilization is widely viewed as an important therapeutic outcome in NP-C [1,3] and was well represented in our patient on the basis of the disability scale assessments. However, even though the disability scale used here is based on empirical multidisciplinary clinical observations [10], due caution should always be adopted in assessing the clinical meaning of such retrospective analyses – prospective assessments offer much greater objectivity.

The age of this patient and the timescale of follow-up assessments largely precluded a quantitative, objective longitudinal analysis of her cognitive function. However, while she displayed early signs of serious cognitive impairment before therapy, her concentration and responsiveness improved after starting miglustat, and her overall cognitive function appeared intact even after periods of frequent epileptic episodes. The observed effects of miglustat on neurological and cognitive function were of great importance to her parents and other caregivers.

It seems clear that intercurrent illnesses require rigorous treatment in parallel with disease-specific therapy in order to maintain quality of life in patients with late-infantile-onset NP-C. Our patient’s repeated respiratory infections and ensuing breathing difficulties had an important impact on her everyday well-being. She also experienced pronounced overproduction of mucus, which was successfully alleviated by using scopolamine skin patches together with saline inhalations and anti-asthmatic therapy.

Seizures are common among patients with late-infantile- and juvenile-onset NPC, but the type, progression, and response to therapy vary considerably. It is important to distinguish between cataplectic and epileptic episodes in order to define the most appropriate therapy. The recurrent bouts of seizure activity in our patient were believed to contribute strongly to significant losses of function during the periods of frequent epileptic attacks.

The precise processes underlying disease progression in NP-C are not yet entirely clear, and it is beyond the scope of this article to assess possible relationships between seizure pathology and NP-C severity. We believe that changes in seizure activity in this case were the result of NP-C disease progression. Seizures numbering more than 50 per day in one period and culminating in full status epilepticus in another had a devastating impact on her overall function and NP-C disability scale score. The successful control of our patient’s seizures by using anti-epileptic therapy resulted in a significant improvement and a return to a level of function that, in our opinion, reflected the true deterioration related to the NP-C disease process. The triple regimen of anti-epileptic therapy that we applied appeared to provide satisfactory and prolonged seizure control. Given this case experience, the control of seizure activity appears vital for the maintenance of overall health and function in NP-C and should be a key focus in clinical management.

## Conclusions

Miglustat appears to have provided distinct therapeutic benefits and improved quality of life in this patient with late-infantile-onset NPC. The effect of miglustat on neurological function and cognition was also vital for the family’s quality of life, as expressed by her parents. This patient’s history highlights the challenges posed by serious epileptic seizure activity and respiratory disease in the clinical management of NP-C. In particular, it is important to effectively control seizures in order to achieve and maintain neurological stabilization during miglustat therapy.

## Consent

Written informed consent was obtained from the parents of the patient for publication of this case report. A copy of the written consent is available for review by the Editor-in-Chief of this journal.

## Abbreviations

MRI: Magnetic resonance imaging; NP-C: Niemann-Pick disease type C.

## Competing interests

The authors declare that they have no competing interests.

## Authors’ contributions

As coordinating study investigator and lead author, JS was involved with all stages of the patient’s treatment and the writing of the manuscript. IBH conducted repeated clinical assessments of the patient at Oslo University Hospital, Norway. BT, also in Oslo, contributed repeated MRI analyses and diagnostic advice. Both co-authors contributed to the written content of the manuscript during draft stages. All authors read and approved the final manuscript.
